# Supplementary material for: Circulating Neutrophils Do Not Predict Subclinical Coronary Artery Disease in Women with Former Preeclampsia
Source: Cells. 2020 Feb 18;9(2):468. doi: 10.3390/cells9020468 (PMC7072843; doi:10.3390/cells9020468)
Supplement: Supplementary file 1 [file cells-09-00468-s001.zip › Supplemental material_Neutro_Crew_R1.pdf]

Supplemental material

Supplemental table 1: Flow cytometry antibodies

Marker	Alternative name	Fluorochrome	Clone	Supplier	Amount (μl)
CD182	CXCR2	AF488	5E8	Biolegend	0.5
CD35	CR1 or C3b/C4b	PE	E11	BD biosciences	5
CD16	FcγRIII	ECD	3G8	Beckman Coulter	0.5
CD14		RPE/Cy7	Rmo52	Beckman Coulter	2.5
CD66b	CEACAM8	APC	80H3	Beckman Coulter	1
CD62L	L-selectin	AF700	DREG56	BD biosciences	0.5
CD11b	CR3A	APC-eFluor 780	ICRF44	eBioscience	2.5
CD184	CXCR4	BV421	12G5	BD biosciences	5
CD9	TSP-29	BV510	M-L13	BD biosciences	5

CD indicates cluster of differentiation; CXCR, C-X-C motif receptor; CR, complement receptor; FcγR, Fc gamma receptor; CEACAM, carcinoembryonic antigen-related cell adhesion molecules; TSP-29, tetraspanin protein 29; AF, Alexa Fluor; PE, (R-) phycoerythrin; ECD, phycoerythrin-Texas Red conjugate (energy coupled dye); RPE/Cy7, (R-) Phycoerythrin: Cy-7 Tandem Conjugate; APC, Allophycocyanin; BV, Brilliant Violet.

Supplemental table 2

		CAC				CAC		
	% cells	No (n=57)	Yes (n=33)	*p*	MFI	No (n=57)	Yes (n=33)	*p*
**CD35+**	Resting	1.9 [1.1-3.4]	1.7 [1.0-3.2]	0.60	Resting	8.2 [8.0-8.4]	8.2 [8.0-8.5]	0.79
	Stimulated	97.0 [93.7-98.5]	97.5 [91.4-98.9]	0.79	Stimulated	15.2 [13.7-16.4]	14.8 [13.0-16.6]	0.72
**CD11B+**	Resting	1.3 [0.7-2.6]	0.9 [0.5-1.5]	0.16	Resting	10.8 [10.5-11.2]	10.6 [10.5-10.9]	0.39
	Stimulated	99.8 [99.3-99.8]	99.7 [99.4-99.8]	0.64	Stimulated	29.4 [25.2-32.8]	27.9 [23.8-32.1]	0.39
**CD66B+**	Resting	0.9 [0.4-1.9]	1.0 [0.6-2.7]	0.20	Resting	20.2 [19.9-20.5]	20.1 [19.9-20.3]	0.21
	Stimulated	49.2 [42.6-60.0]	54.2 [42.8-64.1]	0.41	Stimulated	24.6 [23.4-26.2]	25.2 [23.4-26.4]	0.80
**CD62L-**	Resting	1.4 [0.5-2.1]	0.7 [0.4-3.0]	0.50	Resting	7.2 [4.8-7.9]	7.1 [5.1-7.8]	0.89
	Stimulated	56.9 [48.2-64.7]	54.2 [46.8-64.0]	0.25	Stimulated	2.1 [1.9-2.4]	2.0 [1.8-2.6]	0.72

Supplemental table 2 - Neutrophil activity in women with a history of PE with or without CAC. Neutrophil activity is shown as the percentage or the MFI of cells positive for CD35, CD11B or CD66B and negative for CD62L while resting and upon stimulation with fMLF. Neutrophil activity was not different between women with and without CAC. CAC indicates coronary artery calcification, defined as >0 Agatston Units.

Supplemental table 3

	% cells	Luminal stenosis 0% (n=46)	>0% >50 % (n=35)	≥50% (n=9)	*p*	MFI	Luminal stenosis 0 % (n=46)	>0% >50 % (n=35)	≥50% (n=9)	*p*
**CD35+**	Resting	1.9 [1.1-4.0]	1.7 [1.2-3.2]	1.2 [0.9-2.2]	0.79	Resting	8.2 [8.0-8.4]	8.2 [7.9-8.4]	8.2 [8.0-8.6]	0.99
	Stimulated	97.1 [93.5-98.7]	97.5 [93.2-98.8]	94.2 [90.0-96.9]	0.32	Stimulated	15.3 [13.7-16.8]	15.4 [13.7-16.8]	12.8 [12.1-14.6]	**0.03**
**CD11B+**	Resting	1.4 [0.7-2.5]	0.9 [0.5-1.5]	1.3 [0.4-5.1]	0.14	Resting	10.8 [10.5-11.0]	10.8 [10.5-11.1]	10.6 [10.5-10.8]	0.74
	Stimulated	99.8 [99.5-99.8]	99.7 [99.2-99.8]	99.6 [99.5-99.8]	0.48	Stimulated	29.7 [25.3-32.8]	28.6 [24.4-31.5]	24.6 [22.7-29.6]	0.31
**CD66B+**	Resting	0.9 [0.5-1.9]	1.0 [0.6-2.1]	0.9 [0.6-3.6]	0.67	Resting	20.2 [19.9-20.5]	20.1 [19.8-20.3]	20.2 [20.1-20.4]	0.16
	Stimulated	48.9 [44.1-59.8]	57.4 [41.7-64.2]	47.5 [41.7-51.6]	0.42	Stimulated	24.5 [23.4-26.1]	25.5 [23.5-26.7]	23.6 [23.1-24.5]	0.16
**CD62L-**	Resting	1.4 [0.5-2.1]	1.2 [0.4-2.5]	0.7 [0.5-2.6]	0.70	Resting	6.4 [4.8-7.8]	7.2 [4.6-7.9]	7.6 [6.8-8.0]	0.41
	Stimulated	58.1 [49.1-65.4]	51.5 [46.0-56.9]	64.4 [55.2-67.1]	**0.01**	Stimulated	2.2 [1.9-2.4]	2.0 [1.8-2.2]	2.4 [2.0-2.7]	0.08

Supplemental table 3 – Association of neutrophil activity to degree of luminal coronary stenosis. Neutrophil activity is shown as the percentage or the MFI of cells positive for CD35, CD11B or CD66B and negative for CD62L while resting and upon stimulation with fMLF. In women with ≥50% coronary stenosis, the neutrophil CD35 surface expression (MFI) was lower upon stimulation with fMLF. A similar trend was observed for degranulation marker CD11B, but not for CD66B and CD62L. Indicated *p*-values were calculated with the Kruskal-Wallis test.

Supplemental table 4

		Subclinical CAD				Subclinical CAD		
	**% cells**	**No (n=78)**	**Yes (n=12)**	***p***	**MFI**	**No (n=78)**	**Yes (n=12)**	***p***
**CD35+**	Resting	1.9 [1.1-3.3]	1.2 [1.0-2.6]	0.56	Resting	8.2 [8.0-8.4]	8.2 [7.9-8.5]	0.98
	Stimulated	97.3 [93.3-98.8]	95.4 [91.8-97.8]	0.17	Stimulated	15.3 [13.7-17.0]	13.7 [12.5-14.8]	**0.03**
**CD11B+**	Resting	1.1 [0.7-2.0]	1.0 [0.5-2.3]	0.66	Resting	10.8 [10.5-11.1]	10.6 [10.5-10.9]	0.57
	Stimulated	99.8 [99.4-99.8]	99.7 [99.5-99.8]	1.00	Stimulated	29.2 [25.1-32.8]	26.0 [23.2-29.9]	0.29
**CD66B+**	Resting	0.9 [0.5-1.9]	1.1 [0.6-3.1]	0.32	Resting	20.2 [19.9-20.4]	20.1 [19.9-20.2]	0.97
	Stimulated	50.8 [42.5-63.0]	49.7 [44.9-60.0]	0.88	Stimulated	24.8 [23.4-26.6]	24.1 [23.2-25.2]	0.32
**CD62L-**	Resting	1.3 [0.4-2.4]	0.6 [0.4-2.8]	0.32	Resting	7.2 [4.7-7.9]	6.9 [6.2-7.8]	0.66
	Stimulated	56.1 [48.2-64.5]	57.4 [47.6-65.7]	0.79	Stimulated	2.1 [1.9-2.4]	2.0 [1.8-2.6]	0.92

Supplemental table 4 – Association of neutrophil activity with subclinical CAD. Neutrophil activity is shown as the percentage or the MFI of cells positive for CD35, CD11B or CD66B and negative for CD62L while resting and upon stimulation with fMLF. In women with subclinical CAD, the neutrophil CD35 surface expression (MFI) was lower upon stimulation with fMLF. A similar trend was observed for degranulation marker CD11B, but not for CD66B and CD62L. CAD indicates subclinical coronary artery disease defined as ≥100 Agatston Units and/or ≥ 50 % stenosis. Indicated *p*-values are derived from the Mann-Whitney *U* test.
