# Supplementary material for: Circulating Neutrophils Do Not Predict Subclinical Coronary Artery Disease in Women with Former Preeclampsia
Source: Cells. 2020 Feb 18;9(2):468. doi: 10.3390/cells9020468 (PMC7072843; doi:10.3390/cells9020468)
Supplement: Supplementary file 1 [file cells-09-00468-s001.zip › Supplementary list of QoH and Crew researchers.pdf]

Supplementary material

Circulating neutrophils do not predict subclinical coronary artery disease in women with former preeclampsia

John A.L. Meeuwsen.[1]; Judith de Vries.[1]; Gerbrand A. Zoet[2]; Arie Franx[2], Bart C.J.M. Fauser[3]; Angela H.E.M. Maas[4], Birgitta K. Velthuis[5]; Yolande E. Appelman[6,] Frank L. Visseren[7]; Gerard Pasterkamp[8]; Imo E. Hoefer[8]; Bas B. van Rijn[2]; Hester M. den Ruijter[1]; Saskia C.A. de Jager[1,9]

1. Laboratory for Experimental Cardiology, University Medical Center Utrecht, the Netherlands. 2. Wilhelmina Children's Hospital Birth Center, University Medical Center Utrecht, the Netherlands. 3. Department of Reproductive Medicine & Gynaecology, University Medical Center Utrecht, the Netherlands. 4. Department cardiology, Radboud University Medical Center, Nijmegen, the Netherlands. 5. Department of Radiology, University Medical Center Utrecht, the Netherlands. 6. Amsterdam University Medical Centre, Location VU Medical Centre, VU University, Amsterdam, The Netherlands. 7. Department of Vascular Medicine, University Medical Center Utrecht, the Netherlands. 8. Central Diagnostic Laboratory, University Medical Center Utrecht, Utrecht, the Netherlands. 9. Center Translational Immunology, University Medical Center Utrecht, Utrecht, the Netherlands.

Appendix 1: collaborators of the Queen of Hearts consortium (in alphabetical order)

F.W. Asselbergs[1], E.A.L. Biessen[2], M.L. Bots[3], C. Cheng[4], M.M. Faas[5], C. Ghossein-Doha[6], A.W. Hoes[7], J. Kuiper[8], Y.M. Pinto[9], T. Plösch[10], F.H. Rutten[11], S.A. Scherjon[10], M.A.E. Spaanderman[6], M.C. Verhaar[4], A.J. van Zonneveld[12]

1 Department of Cardiology, Division Heart and Lungs, University Medical Centre Utrecht, Utrecht University, Utrecht, the Netherlands; 2. Experimental Vascular Pathology Group, Department of Pathology, CARIM, Maastricht University Medical Center, the Netherlands; 3. Julius Center for Health Sciences and Primary Care, University Medical Center Utrecht, Utrecht University, Utrecht, the Netherlands; 4. Department of Nephrology and Hypertension, DIGD, UMC Utrecht, University of Utrecht, 3584 CX Utrecht, the Netherlands; 5. Division of Medical Biology, Department of Pathology and Medical Biology, University Medical Center Groningen, University of Groningen, Groningen, the Netherlands; 6 Department of Obstetrics and Gynaecology, Maastricht University Medical Centre, Maastricht, the Netherlands; 7. Julius Center for Health Sciences and Primary Care, UMC Utrecht, Utrecht, the Netherlands; 8. Leiden Academic Center for Drug Research, Leiden University, Leiden, the Netherlands; 9. Heart Center Department of Clinical and Experimental Cardiology Amsterdam Cardiovascular Sciences

Amsterdam University Medical Center University of Amsterdam Amsterdam the Netherlands; 10. Department of Obstetrics and gynaecology, University Medical Center Groningen, University of Groningen, Groningen, Netherlands; 11. Department of General Practice, Julius Center for Health Sciences and Primary Care, University Medical Center (UMC) Utrecht, Utrecht, The Netherlands; 12. Department of Nephrology, Leiden University Medical Center, Leiden, The Netherlands.

Appendix 2: collaborators of the CREW consortium (in alphabetical order)

S.J.Baart[1,2], L. Benschop, MD[3] E. Boersma[4], L. Brouwers[2,5], R.P.J. Budde[4,6], S.C. Cannegieter[7], V. Dam[2,5], M.C.J. Eijkemans[8], M.D. Ferrari[7], Y. van der Graaf[8,] C.J.M. de Groot[9], M.N.Gunning[2,5], A. Hoek[10], H. Koffijberg[8,11], M. Kruit[7], G.R. Lagerwij[2,8], C.B. Lambalk[1], J.S. Laven[1], K.M. Linstra[1,2,8], A. van der Lugt[1], A. Maassen van den Brink[1], C. Meun[1,2], S. Middeldorp[12], K.G.M.Moons[8], J.E. Roeters van Lennep, MD, PhD[13] J.W. Roos-Hesselink[1,] L.J.J. Scheres[2,8,12], E.A.P. Steegers[3], R.P.M. Steegers-Theunissen[1], G.M. Terwindt[7], M.J.H. Wermer[7].

1 Erasmus University Medical Center, Rotterdam, NL; 2 Netherlands Heart Institute, Utrecht, NL; 3 Department of Obstetrics & Gynaecology, Erasmus Medical Center, Rotterdam, NL, 4 Department of Cardiology, Erasmus Medical Center, Rotterdam, NL; 5 University Medical Center Utrecht, Utrecht, NL; 6 Department of Radiology, Erasmus Medical Center, Rotterdam, NL; 7 Leiden University Medical Center, Leiden, NL; 8 Julius Center, Utrecht, University Medical Center, Utrecht, NL; 9 Department of Obstetrics and Gynaecology, VU University Medical Center, Amsterdam, NL; 10 University Medical Center Groningen, Groningen, NL; 11 University of Twente, Enschede, NL; 12 Amsterdam Medical Center, Amsterdam, NL. 13 Department of Internal Medicine, Erasmus Medical Center, Rotterdam, NL
